# Marketing awareness, professional experience, and support for e-cigarette advertising regulation among polish legal professionals: a latent-class analysis

**DOI:** 10.13075/ijomeh.1896.02737

**Published:** 2026

**Authors:** Karolina Zajdel, Anna Merecz-Sadowska, Arkadiusz Sadowski, Piotr Wojtysiak, Dorota Kaleta

**Affiliations:** 1 Medical University of Łódź, Department of Public Health, Łódź, Poland; 2 University of Łódź, Department of Economic and Medical Informatics, Łódź, Poland; 3 Medical University of Łódź, Department of Hygiene and Epidemiology, Łódź, Poland

**Keywords:** regulation, latent class analysis, public policy, advertising, e-cigarettes, marketing awareness

## Abstract

**Objectives:**

To examine whether marketing awareness – operationalized as recognition of e-cigarette advertising persuasiveness – relates to support for advertising regulation among Polish legal professionals, and to identify sociodemographic and occupational correlates of regulatory attitudes.

**Material and Methods:**

Cross-sectional survey of 471 legal professionals in Poland. Latent class analysis identified 3 marketing awareness segments based on 5 perception indicators. The 3-class solution (Bayesian information criterion = 3121.23, entropy = 0.941) distinguished low (38.5%), moderate (47.0%), and high (14.5%) awareness groups. Binary logistic regression modeled support for advertising regulation (“yes” vs. “not-yes”), with awareness class membership and covariates (age, experience, sector, profession) as predictors.

**Results:**

The relationship between marketing awareness and regulatory support could not be established (moderate awareness: odds ratio [OR] = 1.67, 95% CI: 0.99–2.81, p = 0.054; high awareness: OR = 1.82, 95% CI: 0.85–3.90, p = 0.125). Professional experience >10 years (OR = 3.22, 95% CI: 1.02–10.20, p = 0.047) and corporate sector employment (OR = 0.53, 95% CI: 0.30–0.94, p = 0.030) were the only significant predictors.

**Conclusions:**

This exploratory study could not determine whether marketing awareness relates to regulatory support among legal professionals. Professional experience emerged as the primary correlate, suggesting regulatory attitudes may be shaped by professional socialization. Policy efforts should prioritize enforcement of existing restrictions and professional education for early-career practitioners.

## Highlights

- Three awareness classes were identified: low (38.5%), moderate (47.0%), and high (14.5%).
- Marketing awareness did not significantly predict regulatory support.
- Experience >10 years (OR = 3.22) and corporate sector (OR = 0.53) predicted support.

## INTRODUCTION

Electronic-cigarette (e-cigarette) marketing operates across retail, digital, and influencer ecosystems. Converging evidence shows that these activities shape perceived harm, product appeal, and use – particularly among younger audiences and young adults [1–4]. Experimental and longitudinal studies demonstrate that exposure to e-cigarette promotions is associated with lower perceived risk, greater curiosity, and progression toward trial or use [1, 2]. This poses a policy challenge: how to communicate potential harm-reduction benefits for adults who smoke while protecting nicotine-naïve populations from persuasive tactics that normalize vaping and downplay risk [4].

Digital channels are central to this challenge. Content analyses document persistent marketing on social media and email, including discounting, flavor promotion, brand community features, and inconsistent warning placement [5, 6]. Mixed-methods work on TikTok reveals that vaping is often embedded in lifestyle-oriented content [7], while brand-owned feeds sometimes amplify youth-salient themes or blur compliance boundaries [8]. Randomized experiments show that influencer-delivered content can lower perceived harm, whereas simple financial disclosures have limited dampening effects; salient health warnings reduce appeal more effectively [9, 10]. Eye-tracking studies further demonstrate that young adults allocate more visual attention to attractive advertising features than to warnings, and these attention patterns predict favorable attitudes [11, 12]. Together, these findings suggest that individuals responsive to persuasive cues may be less likely to attend to corrective information in the same message environment.

Context-specific evidence underscores the policy relevance for Poland. The Act on the Protection of Health Against the Consequences of Using Tobacco and Tobacco Products [13] prohibits advertising, promotion, and sponsorship of e-cigarettes containing nicotine, with restrictions paralleling those for conventional tobacco products. However, the effectiveness of advertising restrictions depends critically on enforcement capacity. Despite existing legal prohibitions, e-cigarette marketing persists through digital channels, influencer content, and cross-border online sales that are difficult to monitor and prosecute. Legal professionals play a key role in this enforcement ecosystem – as prosecutors, regulatory advisors, and policy advocates – making their attitudes toward advertising restrictions directly relevant to implementation effectiveness. Nationally representative data show that contact with e-cigarette advertising remains common among Polish adolescents and is associated with ever and current use, with nightlife venues and the internet as prominent exposure settings [14]. Although most research focuses on youth and young adults, understanding how adults – particularly professionals who shape policy implementation – interpret e-cigarette marketing and evaluate advertising restrictions is crucial for effective regulation.

Public appetite for stronger advertising controls appears substantial across high-income countries. Cross-national surveys consistently document majority support for e-cigarette advertising restrictions, including bans on broadcast and online advertising, mandatory health warnings, and limitations on youth-targeted marketing [15, 16]. Support levels tend to be higher among non-users, those who perceive e-cigarettes as harmful, and individuals with greater awareness of marketing tactics [15, 17]. Notably, exposure to e-cigarette advertising itself may shape policy attitudes: studies suggest that greater exposure to marketing is associated with more favorable perceptions of e-cigarettes and, paradoxically, lower support for regulatory restrictions [17]. This pattern underscores a potential feedback loop whereby marketing exposure simultaneously increases product appeal and diminishes receptivity to protective policies. Furthermore, message framing can have heterogeneous effects across population subgroups – what Niederdeppe and colleagues term a potential “policy paradox,” wherein warning strategies effective for one group may yield different or even countervailing responses in another [18].

Most empirical studies operationalize “marketing influence” via exposure or recognition, but fewer examine marketing awareness – i.e., a patterned propensity to notice, be persuaded by, or avoid marketing – derived from multiple indicators. Latent class analysis (LCA) provides a principled approach for summarizing multivariate response patterns into probabilistic subgroups, enabling both categorical segmentation and continuous posterior scores for regression models [19, 20]. Applying LCA to advertising-perception items allows examination of how marketing awareness relates to policy attitudes while adjusting for sociodemographic and occupational factors.

The theoretical relevance of marketing awareness extends beyond individual consumer protection. The Persuasion Knowledge Model [21] suggests that individuals who recognize persuasive intent develop coping mechanisms that may translate into support for protective policies. Furthermore, the third-person effect theory [22] proposes that individuals often perceive media effects as stronger on others than on themselves, which may motivate support for regulation to protect vulnerable populations. For legal professionals specifically, understanding how they perceive marketing tactics is policy-relevant because this group interprets and enforces existing tobacco control legislation, potentially shaping regulatory implementation.

Building on this literature, the present study investigates how e-cigarette advertising awareness relates to support for regulation among legal professionals in Poland. The theoretical basis involves competing hypotheses. Greater recognition of persuasive tactics could stem from higher exposure, potentially leading to desensitization or strengthening personal-liberty concerns, predicting lower regulatory support. Conversely, heightened ability to recognize these tactics may reflect critical awareness of marketing's influence on vulnerable populations, predicting greater support for protective policies. This study tests these competing pathways by examining the association between professionals' recognition of advertising awareness and their regulatory attitudes, while adjusting for sociodemographic and occupational factors.

## MATERIAL AND METHODS

### Study design and participants

The authors conducted an anonymous, cross-sectional online survey of legal professionals in Poland during the period March 15 – May 31, 2024. Legal professionals were selected because they directly influence policy implementation through interpretation and enforcement of tobacco control legislation.

Eligible respondents were aged 20–65 years, resided in Poland, and met at least one of the following criteria:

– held a professional legal qualification (barrister, solicitor, notary, judge, prosecutor, or bailiff),
– were enrolled in a formal post-graduate legal traineeship program,
– were employed in a legal role with a completed law degree.

The sample included 461 respondents (97.9%) with a completed master's degree in law and 10 respondents (2.1%) in the final stages of their law degree. The “no completed traineeship” category (N = 134, 28.5%) comprised individuals who had completed legal education but had not yet begun or completed professional traineeship, including those working in legal departments, public administration, or academic positions. In regression analyses, this group is labeled “trainee” to distinguish it from qualified professionals. The “higher education” sector (N = 19, 4.0%) primarily represented doctoral candidates and academic staff.

Exclusion criteria included employment in the nicotine/tobacco industry, inability to provide informed consent, and surveys with missing items. A total of 471 respondents provided complete data for the LCA and regression analyses.

### Sampling, recruitment, and data collection

The authors employed a pragmatic non-probability sampling approach combining outreach through professional associations (national and regional), employer mailing lists, and closed professional social-media groups. The Polish-language instrument was administered via an online survey platform. Invitation materials contained a brief study description and a unique single-use survey link. Unique links prevented duplicate submissions. Respondents provided electronic consent on the landing page and could skip non-mandatory items. No incentives were offered. The final dataset was de-identified, exported to CSV format, and version-controlled using an *a priori* data dictionary.

### Measures

The primary outcome – support for stricter e-cigarette advertising regulation – was assessed with a single item: “Should e-cigarette advertising be regulated more strictly in Poland?” Responses were recorded on a 3-category scale (yes/no/undecided). For the primary analysis, responses were dichotomized as “yes” vs. “not-yes” (combining “no” and “undecided”) to model clear regulatory support. This operationalization captures general attitudinal favorability toward stricter regulation rather than behavioral intentions or readiness to engage in advocacy or enforcement activities.

Latent class analysis was used to identify patterns of marketing awareness based on respondents' recognition of e-cigarette advertising persuasiveness. The latent class model was estimated using 5 indicators representing marketing awareness across 3 conceptual domains. Emotional impact (E) was assessed through emotional valence of advertisements (E1). Persuasive recognition (PR) included modernity cues (PR2) and social acceptability cues (PR3). Channel evaluation (C) comprised social media persuasiveness (C1) and point-of-sale persuasiveness (C3). Pairwise correlations among these 5 indicators were low (all |ρ| <0.70), satisfying the assumption of local independence. Importantly, these indicators assess recognition and evaluation of advertising persuasiveness rather than personal susceptibility to marketing influence. Higher scores reflect greater awareness that marketing employs persuasive tactics, not necessarily greater likelihood of being personally influenced.

Prespecified covariates included age, professional experience, employment sector, and profession. Table 1 summarizes the domains, items, response scales, and coding for all collected variables.

**Table 1.** Survey items and coding schema in cross-sectional online survey of legal professionals (N = 471) in Poland, March–May 2024

Variable	Question stem (abridged)	Response scale/coding	Use in analysis
Regulatory attitude (primary outcome)			
need to regulate advertising (R1)	“Should e-cigarette advertising be regulated more strictly in Poland?”	3 levels: yes/no/undecided; analysis: “yes” vs. “not-yes” (primary); multinomial sensitivity	outcome
Marketing perceptions			
emotional impact			
E1	“Seeing e-cigarette ads evokes positive feelings”	5-point Likert (1–5)	LCA indicator
persuasive recognition			
PR2	“Ads make vaping seem modern/innovative”	5-point Likert (1–5)	LCA indicator
PR3	“Ads make vaping seem socially acceptable”	5-point Likert (1–5)	LCA indicator
channel evaluation			
C1	“How persuasive are social-media posts about e-cigarettes?”	3-point ordinal (0–2)	LCA indicator
C3	“How persuasive are in-store/PoS displays?”	3-point ordinal (0–2)	LCA indicator
Covariate			
age	–	20–25 years (ref.), 26–30 years, 31–40 years, 41–50 years, >51 years	adjustment
professional experience	–	<2 years (ref.), 2–5 years, 6–10 years, >10 years	adjustment
sector	–	public administration (ref.), private-law company, corporation, higher education, not employed	adjustment
profession	–	barrister, solicitor, notary, judge, prosecutor, bailiff	adjustment

C1 – social media persuasiveness; C3 – point-of-sale (PoS) persuasiveness; E1 – emotional valence; LCA – latent class analysis; PR2 – modernity cue; PR3 – social acceptability cue; R1 – regulatory attitude (primary outcome).

Items E1, PR2, PR3, C1, C3 served as inputs to latent class analysis. R1 (regulatory attitude) was the primary outcome. For LCA indicators, directions were harmonized so that higher values reflect greater awareness.

### Statistics

Latent class analysis was performed on 5 advertising-perception indicators using maximum-likelihood expectation-maximization in R (poLCA package). Models with 2–6 latent classes were estimated, each with 100 random starts and a maximum of 10 000 iterations/model to ensure convergence. Model selection was based on multiple fit indices including Bayesian information criterion (BIC), Akaike information criterion (AIC), and log-likelihood, alongside theoretical interpretability and classification quality assessed through relative entropy, which ranges 0–1, with values >0.80 indicating good class separation. The 3-class solution was selected as it provided optimal balance between parsimony and fit, yielding the lowest BIC (3121.23) and demonstrating superior class separation (entropy = 0.941). This solution identified 3 interpretable segments representing low, moderate, and high marketing awareness. Item-response probabilities – the estimated probability of endorsing each response category for a given indicator, conditional on class membership – were computed to characterize the response patterns distinguishing each class.

Univariate associations between each predictor and regulatory support were first examined using χ^2^ tests and unadjusted odds ratios (OR) with 95% confidence intervals (CI). To examine whether marketing awareness differed across demographic and professional subgroups, 1-way analysis of variance (ANOVA) and non-parametric Kruskal-Wallis tests were conducted on posterior probabilities.

Modal class assignments from the selected LCA model served as categorical predictors in subsequent regression analyses, with the low awareness class as the reference category. Posterior probability reflects the estimated likelihood of belonging to a specific class given an individual's observed response pattern; each respondent was assigned to the class with the highest posterior probability (modal assignment). Binary logistic regression modeled support for stricter advertising regulation (“yes” vs. “not-yes”) as a function of latent class membership (2 dummy variables representing moderate and high awareness relative to low awareness) and prespecified covariates (age, professional experience, employment sector, and profession). Regression coefficients (β), ORs equal exp(β), 95% CI, and 2-sided p-values (α = 0.05) were calculated using Huber-White robust standard errors to account for potential heteroscedasticity. Functional form assumptions were evaluated using restricted cubic splines; linearity was retained as appropriate. Multicollinearity was assessed through variance inflation factors, all of which were <2.0. A joint Wald test evaluated the collective contribution of class membership to regulatory attitudes.

### Ethics

This anonymous, non-interventional online survey collected no patient data or special-category personal data under the General Data Protection Regulation (GDPR) regulations. Consistent with institutional and national guidance for survey research, formal ethics committee approval was not required. Participation was voluntary, and proceeding to the questionnaire constituted informed consent. Data were de-identified and stored on secure servers with restricted access.

## RESULTS

### Respondent characteristics

A total of 471 legal professionals provided complete data for analysis. The sample spanned age bands: 20–25 years (N = 26, 5.5%), 26–30 years (N = 158, 33.5%), 31–40 years (N = 224, 47.6%), 41–50 years (N = 55, 11.7%), and >51 years (N = 8, 1.7%). Professional training status was distributed as follows: barrister traineeship (N = 158, 33.5%), solicitor traineeship (N = 153, 32.5%), no completed traineeship (N = 134, 28.5%), judicial traineeship (N = 13, 2.8%), notarial traineeship (N = 9, 1.9%), prosecutorial traineeship (N = 3, 0.6%), and bailiff traineeship (N = 1, 0.2%). Employment sectors comprised private law firms (N = 304, 64.5%), public administration (N = 71, 15.1%), corporations (N = 70, 14.9%), higher education (N = 19, 4.0%), and not employed (N = 7, 1.5%). Professional experience ranged from <2 years (N = 28, 5.9%), 2–5 years (N = 149, 31.6%), 6–10 years (N = 168, 35.7%), to >10 years (N = 126, 26.8%). Regarding the primary outcome, 369 respondents (78.3%) supported stricter regulation, 41 (8.7%) opposed, and 61 (13.0%) were undecided. Professional experience was significantly associated with regulatory attitude (χ^2^ = 14.66, p = 0.023): respondents with >10 years of experience comprised 29.5% of supporters but only 17.1% of opponents and 16.4% of undecided. The undecided group was characterized by younger age (50.8% aged 26–30 years vs. 30.1% among supporters) and shorter experience (44.3% with 2–5 years vs. 29.5%). Marketing awareness class showed similar distributions across all 3 attitude groups (χ^2^ = 3.42, p = 0.490), with no evidence that awareness level differentiated supporters from opponents or undecided respondents.

### Latent classes and marketing awareness construct

Latent class analysis was performed on 5 indicators capturing recognition of e-cigarette marketing persuasiveness: E1, PR2, PR3, C1, and C3. The 5 indicators exhibited balanced response distributions and low pairwise associations (all |ρ| <0.70), supporting their suitability for mixture modeling.

**Figure 1.**
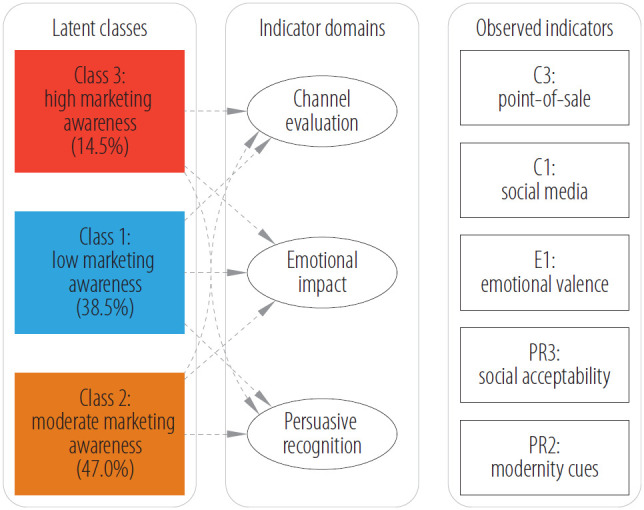
Latent class structure: schematic of the 3-class model showing relationships between latent classes, conceptual domains, and observed indicators in cross-sectional online survey of legal professionals (N = 471) in Poland, March–May 2024

Competing 2- to 6-class solutions were compared using information criteria. The 3-class solution was selected as it minimized the BIC, indicating optimal statistical fit, while offering superior theoretical interpretability and strong classification certainty (entropy = 0.941). This solution yielded 3 interpretable segments reflecting distinct patterns of awareness (Figure 1): class 1 (low awareness, 38.5%, N = 182), class 2 (moderate awareness, 47.0%, N = 222), and class 3 (high awareness, 14.5%, N = 67) (Table 2).

**Table 2.** Latent class analysis (LCA) fit indices and class distribution for the 3-class model in cross-sectional online survey of legal professionals (N = 471) in Poland, March–May 2024

Statistic	Value
LCA fit indices	
indicators [n]	5
Bayesian information criterion	3121.23
Akaike information criterion	3037.76
log-likelihood	–1488.88
relative entropy	0.941
Class distribution [n (%)]	
class 1: low marketing awareness	182 (38.5)
class 2: moderate marketing awareness	222 (47.0)
class 3: high marketing awareness	67 (14.5)

Item-response profiles revealed distinct patterns across classes (Table 3). Class 1 exhibited consistently low probabilities of endorsing high-level responses across all 5 indicators, suggesting limited recognition of marketing persuasiveness. Class 2 showed moderate engagement, with elevated probabilities for mid-level responses on persuasive-recognition and channel-specific indicators. Class 3 displayed the highest probabilities across all domains, particularly for PR3 and C3, indicating the strongest recognition of marketing tactics.

**Table 3.** Item-response probabilities by latent class: conditional probabilities of endorsing each response level across the 5 indicators in cross-sectional online survey of legal professionals (N = 471) in Poland, March–May 2024

Variable	Probability		
	class 1 (N = 182,38.5%)	class 2 (N = 222,47.0%)	class 3 (N = 67,14.5%)
E1			
positive	0.02	0.03	0.01
neutral	0.88	0.66	0.63
negative	0.10	0.31	0.36
PR2			
high appeal	0.17	0.00	0.59
moderate appeal	0.04	0.33	0.00
low appeal	0.78	0.67	0.41
PR3			
acceptable	0.88	0.78	0.94
not acceptable	0.12	0.22	0.06
C1			
noticed actively	0.00	0.00	0.01
noticed passively	0.11	0.11	0.18
not noticed	0.89	0.89	0.81
C3			
high persuasion	0.00	0.22	0.47
moderate persuasion	0.20	0.54	0.53
low persuasion	0.80	0.23	0.00

Class 1 – low awareness; class 2 – moderate awareness; class 3 – high awareness. Other explanations as in Table 1.

Modal class assignments served as categorical predictors in subsequent regression analyses, with class 1 as the reference category. Mean posterior probability of assignment to the modal class was 0.95 with a standard deviation of 0.12, indicating very high classification certainty.

### Marketing awareness distribution across demographic and professional groups

**Figure 2.**
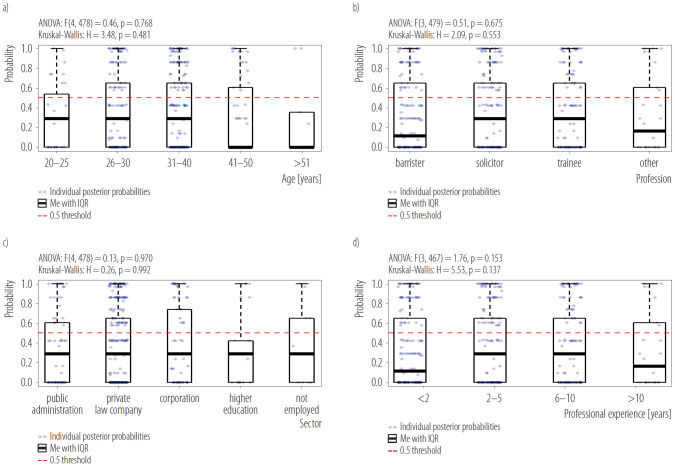
Marketing-awareness distribution across subgroups: a) age, b) profession, c) employment sector, and d) professional experience in cross-sectional online survey of legal professionals (N = 471) in Poland, March–May 2024

**Figure 3.**
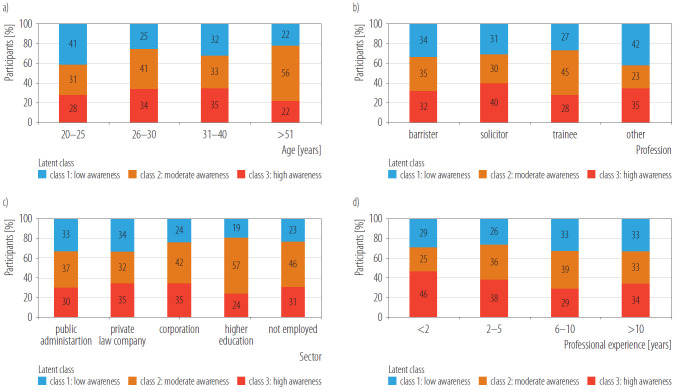
Class composition by subgroup: a) age, b) profession, c) sector, and d) experience in cross-sectional online survey of legal professionals (N = 471) in Poland, March–May 2024.

Marketing awareness probabilities exhibited broadly overlapping distributions across age, profession, sector, and experience strata (Figure 2). Omnibus tests (ANOVA and Kruskal-Wallis) revealed no significant between-group differences (all p > 0.05), indicating that awareness was uniformly distributed throughout the sample. Consistent with these findings, latent class membership was similarly balanced across all demographic and professional categories (Figure 3). Each subgroup contained representatives from all 3 awareness classes, with proportional distributions ranging from 10–20% high awareness, 40–50% moderate awareness, and 35–45% low awareness across strata. These patterns suggest that recognition of marketing persuasiveness was not concentrated in specific demographic or professional segments but rather broadly distributed across the legal professional population.

Omnibus tests (ANOVA and Kruskal-Wallis) showed no significant between-group differences (all p > 0.05). Whiskers extend to the most extreme observed value within 1.5 × IQR of the box; outliers not shown.

### Determinants of support for advertising regulation

Univariate analyses revealed no significant association between marketing awareness class and regulatory support (χ^2^ = 0.67, df = 2, p = 0.715). Support rates were similar across classes: low awareness 80.2%, moderate awareness 77.5%, and high awareness 76.1%. Professional experience showed a significant univariate association (χ^2^ = 8.12, df = 3, p = 0.044), with respondents having >10 years of experience showing the highest support rate (86.5% vs. 71.4% for <2 years; unadjusted OR = 2.56, 95% CI: 0.98–6.74). Age approached significance (χ^2^ = 9.39, p = 0.052), while employment sector (χ^2^ = 2.59, p = 0.629) and profession (χ^2^ = 2.27, p = 0.517) showed no significant univariate associations. Table 4 presents both unadjusted and adjusted estimates. Thus, the absence of association between marketing awareness and regulatory support was evident at both univariate and multivariate levels, indicating that covariate adjustment did not suppress or confound this relationship.

**Table 4.** Binary logistic regression predicting support for stricter e-cigarette advertising regulation (outcome: “yes” vs. “not-yes”) in cross-sectional online survey of legal professionals (N = 471) in Poland, March–May 2024

Predictor and category	uOR	95% CI	β	aOR	95% CI	p
Latent class (ref. low awareness)						
moderate marketing awareness	0.85	0.52–1.37	0.511	1.67	0.99–2.81	0.054
high marketing awareness	0.79	0.40–1.54	0.600	1.82	0.85–3.90	0.125
Age (ref. 20–25 years)						
26–30 years	0.56	0.20–1.58	−0.650	0.52	0.22–1.23	0.136
31–40 years	1.10	0.39–3.08	−0.089	0.92	0.40–2.12	0.840
41–50 years	1.22	0.36–4.08	−0.305	0.74	0.30–1.82	0.507
>51 years	1.67	0.17–16.81	0.025	1.03	0.28–3.77	0.970
Experience (ref. <2 years)						
2–5 years	1.09	0.44–2.67	0.448	1.57	0.75–3.26	0.232
6–10 years	1.42	0.58–3.47	0.711	2.04	0.98–4.25	0.059
>10 years	2.56	0.98–6.74	1.169	3.22	1.02–10.20	0.047*
Sector (ref. public administration)						
private law firm	0.64	0.32–1.28	−0.324	0.72	0.43–1.22	0.226
corporation	0.53	0.23–1.22	−0.637	0.53	0.30–0.94	0.030*
higher education	0.69	0.19–2.46	−0.596	0.55	0.23–1.31	0.179
not employed	1.10	0.12–10.05	0.031	1.03	0.32–3.38	0.956
Profession (ref. barrister)						
solicitor	1.39	0.81–2.38	0.377	1.46	0.83–2.58	0.193
trainee	1.28	0.74–2.22	0.599	1.82	0.98–3.37	0.057
other	1.86	0.61–5.74	0.656	1.93	0.89–4.16	0.095

Joint Wald test for latent class membership: χ^2^(2) = 4.81, p = 0.090.

* p < 0.05 (2-tailed).

In the primary binary logistic regression (N = 471), the relationship between marketing awareness and support for regulation could not be established. Latent class membership showed positive but non-significant associations with regulatory support (moderate awareness: β = 0.511, OR = 1.67, 95% CI: 0.99–2.81, p = 0.054; high awareness: β = 0.600, OR = 1.82, 95% CI: 0.85–3.90, p = 0.125) (Table 4). *Post hoc* power analysis revealed that with N = 471 and α = 0.05, the study achieved 80% power to detect large effects (OR ≥ 2.0) but had insufficient power for small-to-medium effects (OR = 1.5–1.9, power 51–68%).

Among covariates, professional experience and employment sector emerged as significant predictors. Professionals with >10 years of experience showed over 3 times higher odds of supporting regulation compared to those with <2 years (OR = 3.22, 95% CI: 1.02–10.20, p = 0.047). Conversely, employment in the corporate sector was associated with significantly lower odds of support relative to public administration (OR = 0.53, 95% CI: 0.30–0.94, p = 0.030). Age and professional training showed no significant associations (all p > 0.05) (Table 4). These findings suggest that regulatory attitudes are shaped primarily by professional socialization and institutional context rather than individual-level recognition of marketing awareness.

In sensitivity analysis using multinomial logistic regression with 3 outcome categories (yes/no/undecided), marketing awareness class remained non-significant for both contrasts. Compared to low awareness, moderate and high awareness showed no significant association with opposing regulation (OR = 0.96, p = 0.917 and OR = 1.72, p = 0.231, respectively) or being undecided (OR = 1.33, p = 0.338 and OR = 0.95, p = 0.920, respectively). These findings confirm that the null association was not an artifact of outcome dichotomization.

## DISCUSSION

The authors identified 3 latent classes of “marketing awareness” toward e-cigarette advertising – low (38.5%), moderate (47.0%), and high (14.5%) – with strong separation (entropy = 0.941). However, class membership did not significantly predict support for stricter advertising regulation in adjusted models (joint Wald p = 0.090). The only significant correlates of support were >10 years of professional experience (OR = 3.22) and employment in the corporate sector (OR = 0.53). Given limited statistical power for small-to-moderate effects, these results remain inconclusive.

Two considerations help contextualize these findings. First, measurement: the LCA indicators capture recognition that advertising employs persuasive tactics, not personal susceptibility to persuasion. Individuals who critically recognize sophisticated marketing may, paradoxically, also endorse regulation, blurring directional expectations. Second, professional socialization: regulatory attitudes among legal professionals may be driven more by experience-based norms than by individual perceptions of advertising; this aligns with the positive association for >10 years of experience and the lack of systematic differences by age or profession.

The broader evidence base corroborates that e-cigarette marketing can shape perceptions and behavior, particularly among young people. Experimental and longitudinal studies associate exposure with more positive attitudes, reduced perceived harm, and greater trial or use [1, 2]. Digital channels are central: content analyses document frequent discounting, flavor promotion, brand-community appeals, and inconsistent or missing warnings in retailer emails and social-media promotions [5, 6, 8]. Randomized experiments indicate that influencer-delivered content can decrease perceived harm and heighten susceptibility; by contrast, simple financial disclosures have limited dampening effects, whereas prominent health warnings reduce appeal more reliably [9, 10]. Eye-tracking work shows that young adults preferentially attend to attractive features and flavors rather than to warnings, and that attention patterns predict more favorable perceptions and expectancies [11, 12]. In Poland specifically, nationally representative data indicate that contact with e-cigarette advertising is common among adolescents and associated with use, with nightlife venues and the internet as prominent exposure settings [14].

These findings inform the interpretation of the inconclusive awareness–attitude link. In a professional, highly educated sample, recognition of persuasive tactics may reflect media literacy rather than vulnerability and thus be only weakly tied to personal policy preferences. Moreover, heterogeneity in message effects across groups complicates simple predictions: warning framings that are effective for youth may yield different (even countervailing) responses among adults who smoke or vape – the “policy paradox” [18].

The weight of evidence supports prioritizing oversight of influencer and algorithm-amplified marketing. Restrictions on paid influencer promotion of nicotine products, coupled with standardized, high-salience warnings (front-loaded, high-contrast, minimum size), are consistent with experimental data showing weak effects of disclosures but stronger dampening from warnings [9, 10]. Enforcement should extend to retailer-owned channels (email, brand feeds) where warning implementation and placement remain inconsistent and often non-compliant [5, 6]. Given that professional experience rather than awareness predicted support in the authors' sample targeted continuing professional education for early-career legal practitioners could emphasize high-quality summaries of marketing effects on youth and the differential impacts of warning framing [11, 14, 18]. Public sentiment appears receptive to stronger advertising controls, with cross-national evidence documenting majority support for e-cigarette advertising restrictions among both adults and young people [15–17], suggesting a window for policy action.

This cross-sectional study used non-probability sampling of a specific professional group, limiting generalizability; self-selection may have attracted respondents with stronger pre-existing views on tobacco policy. Data were collected (March–May 2024) before the May 2025 amendment that extended advertising restrictions to all e-cigarette products; findings reflect attitudes under the pre-amendment regulatory framework and may not generalize to the current legal context. The cross-sectional design precludes causal inference – the authors cannot determine whether professional experience shapes regulatory attitudes or whether individuals with pro-regulatory attitudes pursue longer legal careers. Social desirability bias may have inflated support rates, as legal professionals might favor responses perceived as socially responsible. Additionally, the authors did not assess personal e-cigarette or tobacco use, which could confound the awareness–attitude relationship.

Regarding methodological choices that may have influenced results, the operationalization of marketing awareness as recognition rather than personal susceptibility may not capture mechanisms most relevant to policy attitudes. The primary analysis dichotomized regulatory support (“yes” vs. “not-yes”); however, sensitivity analysis using multinomial regression with all 3 response categories (yes: 78.3%, no: 8.7%, undecided: 13.0%) confirmed null findings for marketing awareness. Furthermore, analyzed measure captured general attitudinal favorability rather than readiness to act; support may range from passive approval to active willingness to engage in advocacy or enforcement, and these dimensions may relate differently to marketing awareness. The authors note that significant findings for professional experience and sector emerged despite these methodological constraints, indicating the approach was capable of detecting meaningful associations where they existed. Finally, the Polish regulatory context may limit transferability to jurisdictions with different e-cigarette marketing environments. Future studies would benefit from larger samples, longitudinal designs, multi-item regulatory attitude scales, measures of personal tobacco/nicotine use, and replication across professional groups and national contexts.

## CONCLUSIONS

In this professional sample, marketing awareness – operationalized as recognition of persuasive tactics – did not significantly predict support for stricter advertising regulation. Given limited statistical power for small–moderate effects, this finding remains inconclusive. Professional experience (>10 years) was the most consistent correlate of support, with corporate employment associated with lower support. These patterns suggest that professional norms and experience, more than individual recognition of marketing tactics, shape regulatory attitudes in this context.

Policy efforts should prioritize enforcement of e-cigarette advertising restrictions on digital platforms and influencer marketing, and professional associations should incorporate tobacco control evidence into continuing education for early-career practitioners. Regarding enforcement of existing regulations, legal professionals can contribute by prosecuting advertising violations on social media and influencer platforms, advising retailers and e-cigarette companies on compliance with health warning requirements, and engaging in regulatory consultations to address enforcement gaps in cross-border online sales and algorithm-driven marketing. Future research should employ longitudinal designs to determine whether experience causally shapes attitudes, compare awareness–attitude relationships across professional groups involved in policy implementation, and develop multi-item scales measuring both recognition of and susceptibility to marketing.
